# Single center experience in Next Generation Sequencing for genetic diagnosis of Autoinflammatory Disorders (AIDs)

**DOI:** 10.1186/1546-0096-13-S1-P52

**Published:** 2015-09-28

**Authors:** FR Lepri, E Pisaneschi, D Minervino, V Messia, M Pardeo, F de Benedetti, A Insalaco

**Affiliations:** 1 Bambino gesù children hospital, 1Cytogenetics and Molecular Genetics, Rome, Italy; 2 Bambino Gesù Children Hospital, Pediatric Medicine-Rheumatology, Rome, Italy

## Introduction

Autoinflammatory disorders (AIDs) represent an expanding group of complex diseases characterized by periodic or chronic systemic inflammations. Mutations in more than 15 geneshave been associated with autoinflammatory recessive or dominant syndromes. Next Generation Sequencing (NGS) has emerged in the last year as new diagnostic tool in this field.

## Objectives

To share data obtained by the use of NGS in a cohort of patients affected by an autoinflammatory disease of undefined origin evaluated at our center.

## Materials and methods

In this study we enrolled 158 patients from 2010 to 2014. We developed NGS starting with 11 genes already known to be involved in AID (Panel 1: MVK, MEFV, NRLP12, NRLP3, NOD2, TNFRSF1A and PSTPIP1 and Panel 2: IL1RN, LPIN2, IL36RN, PSMB8). Targeted resequencing was performed using customized panel and analyzed with the MiSeq^®^ sequencing platform (Illumina, San Diego, CA). All variants identified have been confirmed by Sanger sequencing.

## Results

48,(73%) of the patients present variants in genes of Panel 1. 32,46% have variants in NLRP3 gene: the most frequent variants are Q705K (56%) and V200M (32%). About 26% have variants in NOD2, the most frequent variant is R702W (25%). 30% have variants in NLRP12, the most frequent variant is F402L (65%), in two cases in homozygosity. 23,4% have variants in MEFV, the most frequent variant is E148Q (22%). 4% have variants in MVK, V377I (100%). 10,38% have variants in TNFRSF1A, the most frequent variant is R121Q (75%). 9% have variants in PSTPIP1. 69% of the patients present variant in only one gene; 28,57% present variants in two different genes and two patients in three genes. We performed 15 familial study to unravel the segregation of some variants.

## Conclusion

NGS leads to the identification of many genetic variants that could be associated with disease susceptibility. The major challenge is in the interpretation of the clinical relevance of identified variants. Some patients show variants in multiple analyzed genes: it can be assumed that different variants in different genes may cooperate to determine a pathological phenotype. This will necessitate large-scale population studies, in vitro functional assay and careful correlation of genetic information with phenotypic data. Therefore, close collaboration with clinicians is crucial.

